# Healthcare-associated infection: do doctors recognize this group of patients?

**DOI:** 10.1186/cc9635

**Published:** 2011-03-11

**Authors:** T Cardoso, O Ribeiro, I Aragão, A Costa-Pereira, A Sarmento

**Affiliations:** 1 Hospital de Santo António, Porto, Portugal; 2 Faculty of Medicine, University of Porto, Portugal; 3 Hospital de São João, Porto, Portugal

## Introduction

Traditionally infections are divided into community acquired (CAI) or hospital acquired (HAI). The authors study the association between healthcare-associated infections (HCAI) and inappropriate antibiotic therapy and hospital mortality.

## Methods

A prospective cohort study (1 year), in five wards of a university hospital, including all consecutive adult patients that met the CDC definition of infection. They were classified in: CAI, HCAI (using Friedman's classification [1]) and HAI. A multivariable logistic regression was used with inappropriate antibiotic therapy as the dependent variable and sex, age, previous co-morbidities, type of infection (CAI, HCAI or HAI), severity of infection, SAPS II, total SOFA score, focus of infection, polymicrobial infection, previous antibiotic therapy, positive blood cultures, number of hospitalizations in the previous year and Karnovsky index as independent variables, and a similar model with also inappropriate antibiotic therapy and microbiological diagnosis with hospital mortality as the dependent variable.

## Results

We included 1,035 patients: 493 (48%) with CAI, 225 (22%) with HCAI and 317 (31%) with HAI. HCAI (adjusted OR = 1.905, 95% CI = 1.152 to 3.152) was associated with inappropriate antibiotic therapy. The following variables were associated with hospital mortality: HAI (adjusted OR = 2.095, 95% CI = 1.275 to 3.441), cancer (adjusted OR = 2.768, 95% CI = 1.316 to 5.823), diabetes (adjusted OR = 0.420, 95% CI = 0.228 to 0.775), Karnovsky index (adjusted OR = 0.968, 95% CI = 0.958 to 0.978), SAPS II (adjusted OR = 1.107, 95% CI = 1.085 to 1.128) and inappropriate antibiotic therapy (adjusted OR = 1.663, 95% CI = 1.006 to 2.747). HCAI was not associated with increased hospital mortality (adjusted OR = 0.808, 95% CI = 0.449 to 1.453), although this group of patients had higher SAPS II (median = 30 vs. 28 in the other two groups, *P *= 0.002), no differences were found regarding median SOFA score or severity of infection.

## Conclusions

HCAI was not associated with increased hospital mortality but it was associated with inappropriate antibiotic therapy, an independent prognostic factor. Doctors might not be sufficiently aware of this new group of patients. Locally driven information campaigns are needed.
